# A System of Practical Medicine, by American Authors

**Published:** 1886-09

**Authors:** 


**A System of Practical Medicine by American Authors.** Edited by WM. PEPPER, M. D., LL. D., Provost and Professor of Theory and Practice of Medicine in the University of Pennsylvania; assisted by LOUIS STARR, M. D., Clinical Professor of Diseases of Childen. Vol. v. Diseases of the Nervous system. Philadelphia: Lea Brothers & Co. 1886.

This, the last volume of this great work, is devoted entirely to the consideration of the diseases of the nervous system; and, in its 1,300 pages, the various functional and organic derangements of the nervous system are exhaustively treated by such distinguished authors as Seguin, Charles K. Mills, H. C. Wood, Weir Mitchell, McLane Hamilton, Spitzka and others. Articles from such authors need no commendation, and space forbids our noticing, at length, this admirable book; it is sufficient to say that it is in every way worthy of a place with the other volumes of the American system of medicine.

The appearance of the successive volumes has called forth the highest praise from both American and European journals, and now the entire work is completed we cannot refrain from again expressing our warm admiration. We look upon it as one of the greatest works on practical medicine in the English language, and a glory to American medicine. Possibly there may be a few Teutonophilists who assert that there are translations of German works which are superior, but the vast majority of the profession will, we believe, agree with our opinion. Its 6,000 pages embraces the whole domain of medicine, with the exception of midwifery, and matters strictly surgical, and it may be truly regarded as a "complete library of practical medicine," and the general practitioner will require little else in the daily round of professional duties.

